# *Ganoderma* Fusions with High Yield of Ergothioneine and Comparative Analysis of Its Genomics

**DOI:** 10.3390/jof9111072

**Published:** 2023-11-02

**Authors:** Jiaqi Xie, Yinghao Yu, Junjiang You, Zhiwei Ye, Fenglong Zhou, Na Wang, Jingru Zhong, Liqiong Guo, Junfang Lin

**Affiliations:** 1 College of Food Science, South China Agricultural University, Guangzhou 510640, China; xiejiaqi0524@gmail.com (J.X.); yuyinghao1995@stu.scau.edu.cn (Y.Y.); youjacob1364869718@outlook.com (J.Y.); zhoufenglong2023@outlook.com (F.Z.); guolq@scau.edu.cn (L.G.); 2 Research Center for Micro-Ecological Agent Engineering and Technology of Guangdong Province, Guangzhou 510640, China; 3 Guangzhou Alchemy Biotechnology Co., Ltd., 139 Hongming Road Guangzhou Economic Technology Zone, Guangzhou 510760, China; wangn@ajfz.com.cn (N.W.); jameszhong8999@gmail.com (J.Z.)

**Keywords:** *Ganoderma* fusions, protoplast fusion, ergothioneine, genome analysis

## Abstract

Ergothioneine (EGT), an exceptional antioxidant found ubiquitously across diverse living organisms, plays a pivotal role in various vital physiological regulatory functions. Its principal natural sources are mushrooms and animal liver tissues. *Ganoderma* spp., a traditional Chinese food and medicinal mushroom, boasts high concentrations of EGT. To advance the development of novel *Ganoderma* spp. strains with enhanced EGT yields, we employed an efficient *Ganoderma* spp. protoplasmic fusion system. Through molecular and biological characterization, we successfully generated seven novel fusion strains. Notably, fusion strain RS7 demonstrated a remarkable increase in mycelial EGT production (12.70 ± 1.85 mg/L), surpassing the parental strains FQ16 and FQ23 by 34.23% and 39.10%, respectively. Furthermore, in the context of the fruiting body, fusion strain RS11 displayed a notable 53.58% enhancement in EGT production (11.24 ± 1.96 mg/L) compared to its parental strains. Genomic analysis of the RS7, the strain with the highest levels of mycelial EGT production, revealed mutations in the gene EVM0005141 associated with EGT metabolism. These mutations led to a reduction in non-productive shunts, subsequently redirecting more substrate towards the EGT synthesis pathway. This redirection significantly boosted EGT production in the RS7 strain. The insights gained from this study provide valuable guidance for the commercial-scale production of EGT and the selective breeding of *Ganoderma* spp. strains.

## 1. Introduction

Tanret [1] originally isolated a distinctive sulfur-containing compound from *Claviceps purpurea*, subsequently identified as 2-thiol-L-histidine trimethyl betaine, and named it ergothioneine (EGT). EGT is a colorless and odorless compound with a molecular weight of 229.30 g/mol. In solution, it presents as a dynamic equilibrium between thiol and thione isomeric structures [2], mainly adopting the thione form at a physiological pH. EGT is ubiquitously distributed in plants, humans and other animal organisms, where it serves as a pivotal role. Notably, EGT exhibits elevated baseline concentrations in liver and blood cells, and its accumulation in damaged tissues suggests a potential adaptive cytoprotective mechanism [3]. Despite its prevalence in human tissues, EGT is not currently considered an essential nutrient, and no research has reported adverse symptoms resulting from EGT deficiency [4]. Owing to its potent antioxidant properties and low metabolic consumption rate, EGT is associated with cell protection [5], its antidepressant qualities [6], as well as its role in ocular safeguarding [7] and alleviating cerebrovascular conditions [8]. While the human body cannot synthesize EGT endogenously, it can be obtained from the environment and accumulated through the consumption of mushrooms and animal liver [9].

*Ganoderma* spp., a group of wood-degrading fungi with a hard fruiting body, belongs to the kingdom of Fungi, division of Basidiomycota, class of Homobasidiomycetes, order of Aphyllophorales, family of Polyporaceae (Ganodermataceae) and genus of *Ganoderma* [10]. Although certain strains of *Ganoderma* have been linked to diseases in cash crops [11,12], researchers have also recognized the medicinal potential of some *Ganoderma* strains. In China, *Ganoderma* spp. have served as valuable traditional medicinal resources for over 2000 years, with more than 100 known species within the Ganoderma genus. *Ganoderma* spp. are particularly esteemed for their medicinal attributes, due to their high content of EGT, polysaccharides and triterpenes as well as anti-tumor, hypotensive and immune-enhancing properties [13]. *Ganoderma*-based products have received widespread attention in Europe, Malaysia, North America and Singapore [14], and over 1200 *Ganoderma*-based health food products are currently acknowledged [15]. Earlier studies have highlighted that among dietary sources, edible mushrooms offer the highest EGT levels available [16]. Additionally, Weigand-Heller et al. [17] confirmed the efficient absorption and utilization of EGT from edible mushrooms by the human body post-consumption. Notably, their research identified *Ganoderma* spp. as having the highest EGT levels among all the edible mushrooms examined [18].

Advancements in breeding techniques have brought about significant enhancements and innovations in protoplast fusion, expanding its applications and facilitating the development of novel strains with desirable traits. Protoplast fusion entails the merging of parental protoplasts possessing distinct genetic traits induced by fusion agents. This enables the effective amalgamation of genomes from diverse cells, culminating in the exchange and recombination of genes, thus achieving the fusion of parental genetic characteristics [19]. The main breeding processes include protoplasmic preparation and regeneration, protoplast inactivation and fusion, [20] as well as fusion screening and identification [21]. This technique has been extensively utilized in the breeding of *Ganoderma* and other edible mushrooms. For instance, He et al. [22] demonstrated its application in the breeding of *M. importuna*, Bok et al. [23] performed protoplast fusion between *Shiitake* and *Ganoderma*, and Chiu et al. [24] conducted protoplast fusion between two distinct *Ganoderma* species. Protoplast fusion technology offers distinct advantages, including the ability to surmount the limitations of distant hybridization, enhancement of desirable traits, and broad applicability in the realm of breeding. In our study, we utilized protoplast fusion technology to generate fusion strains by merging the indigenous *Ganoderma* strains, FQ23 and FQ16, both of which exhibit high levels of EGT production. These chosen fusions held the promise of yielding new strains with increased medicinal value and serving as invaluable research resources for future investigations into *Ganoderma* spp. Meanwhile, we selected fusions RS7, RS8 and RS12 with high, medium and low EGT contents, respectively, for genome resequencing. This approach allowed us to dissect the fusion mechanism by conducting genome comparisons, thereby providing theoretical guidance for selective breeding of new *Ganoderma* strains with high ergothioneine production. Our overarching objective was to elevate the overall worth of Ganoderma spp. through the development of strains with improved food safety, EGT yields, and fruiting body quality.

## 2. Materials and Methods

### 2.1. Stains and Culture Maintenance

*G. resinaceum* strain FQ23 and *G. sessile* strain FQ16 were isolated from wild mushrooms collected in Hainan Province and stored in the Food Technology College of South China Agricultural University (Guangzhou, China). Vegetative cultures were propagated on Potato Dextrose Agar (PDA) medium (2.5% glucose, 0.3% KH_2_PO_4_, 0.15% MgSO_4_·7H_2_O, 2% agar in 1 L of filtered boiled potato juice). Mycelia were cultured in Potato Sucrose Broth (PSB) medium (2.5% sucrose, 0.3% KH_2_PO_4_, 0.15% MgSO_4_·7H_2_O and 0.2% NH_4_Cl and 1 L of filtered boiled potato juice). The medium employed for protoplast regeneration consisted of 3 g/L sucrose, 20 g/L glucose and 2 g/L yeast extract, 2 g/L peptone, 3 g/L KH_2_PO_4_, 1.5 g/L MgSO_4_·7H_2_O, 0.004 g/L vitamin B1, 0.6 M Mannitol and 1 L of filtered boiled potato juice.

### 2.2. Preparation and Regeneration of Protoplast

Protoplast preparation and regeneration were carried out according to the method of Wu et al. [25] and adjusted to be suitable. A total of 0.3 g wet mycelia of FQ23 and FQ16, which were cultured in PSB medium for 3–10 days at 28 °C and 150 rpm, were obtained and rinsed with an osmotic stabilizer consisting of 0.6 M D-mannitol. Following this, the mixture was subjected to centrifugation at 10,000 rpm for 5 min, after which the supernatant was discarded.

First, after adding 300 μL of filter-sterilized lywallzyme (Guangdong Institute of Microbiology, Guangzhou, China), the mycelium was ground with a sterilized pestle, and then 700 μL of filter-sterilized lywallzyme was added to dissolve cell wall at 30 °C and 150 rpm for 3.5 h. After enzymatic hydrolysis, the enzyme solution was first filtered out using 6 layers of lens paper to absorb excess water and then transferred into a new 1.5 mL centrifuge tube and subjected to centrifugation at 500 rpm for 10 min. The supernatant was subsequently transferred into a new 1.5 mL centrifuge tube and centrifuged again at 3000 rpm for 10 min. The resulting pellet, consisting of the transformed protoplasts, was collected and the supernatant was discarded. To eliminate residual enzyme solution, the protoplasts were centrifuged after the addition of 0.5 mL of a 0.6 M sucrose solution at 3000 rpm for 10 min. The supernatant was discarded and this process was repeated twice. Subsequently, the sedimented protoplasts were suspended immediately in an osmotic stabilizer solution. The yield of protoplasts (cells/mL^−1^) was quantified using a Neubauer hemocytometer [26]. After counting, the protoplast suspension was divided into two portions, and the number of protoplasts was adjusted to about 10^4^ protoplasts/mL using 0.6 M sucrose solution and sterile water, respectively. In total, 100 μL of protoplasts was aspirated and applied to regeneration plates and PDA plates, and three parallel groups of each parent were set up in each group. After 4 days of incubation at 28 °C, the colonies on regeneration plates and PDA plates were counted. The protoplast regeneration rate (%) was calculated as follows:

$$Protoplast\;regeneration\;rate\left(\%\right)=[\left(Rn-Pn\right)/T]\times 100\%$$

Rn: Number of regeneration medium colonies

Pn: Number of PDA plate colonies

T: Number of protoplasts

### 2.3. Protoplast Inactivation Labeling and Fusion

#### 2.3.1. Parental Protoplast UV-Inactivation Labeling

Taking into account the three indicators of culture time, number of protoplasts and regeneration rate, the mycelium with appropriate age was selected for UV double inactivation to label protoplasts. The concentration of parental protoplasts was adjusted to 10^6^ protoplasts/mL. The protoplast suspension was transferred to a sterile plate and exposed to UV radiation for 90 s and 120 s under dark conditions with a 38 W UV lamp positioned 30 cm away from the plate. Following the irradiation, 100 μL of the protoplast suspension was applied to the regeneration plates. In the regeneration experiment, three groups were set in parallel for each condition, and the irradiation for 0 s was used as the control. After incubation at 28 °C, the irradiation time with an inactivation rate of 100% was selected as the subsequent experimental condition.

#### 2.3.2. Protoplast Fusion and Regeneration

Fusion was conducted according to the method described by Chakraborty and Sikdar [27], with slight modifications. With a 100% inactivation rate after UV inactivation, both the protoplast suspensions of FQ23 and FQ16, which had been already taken at a density of 10^6^–10^7^, were added in equal volumes to the same 1.5 mL centrifuge tube. Subsequently, the tubes were centrifuged at 3000 rpm for 10 min. After discarding the supernatant, the protoplast precipitate was treated with an equivalent volume of 30% PEG reagent as the supernatant. The suspensions were gently mixed and maintained at 30 °C for 30 min. The suspensions were then centrifuged at 3000 rpm for 10 min and washed twice with 1 mL of 0.6 M sucrose solution. Later, the protoplast suspension adjusted to 10^6^ protoplasts/mL was transferred to the regeneration medium. The plates were incubated at 28 °C for observation of the protoplast regeneration and development of colonies.

### 2.4. Fusions Identification

#### 2.4.1. Antagonistic Identification Test

For antagonistic identification test, the hyphae of parents and fusions were inoculated in PDA. Putative fusions capable of producing strong antithetic lines with their parents were obtained by subculture until the fifth generation. After the 5th subculture, fusions that could still produce significant confrontation lines with their parents were preserved.

#### 2.4.2. Identification of Putative Fusons by ISSR-PCR 

The primers (listed in Table 1) with good band polymorphism were screened from 82 ISSR universal primers using 2×pcrmix as identification primers (Synthesized by Guangzhou Tianyi Huiyuan Gene Technology Co., Ltd., Guangzhou, China). Genomic DNA was extracted from the mycelia of parents and fusions passed onto the fifth generation using the method described by Al-Samarrai and Schmid [28]. From the initial screening of 82 ISSR primers, sixteen primers were selected. ISSR amplifications were conducted using a 25 μL reaction mixture containing 12.5 μL of 2×pcrmix (Purchased from Vazyme Biotechnology Ltd., Nanjing, China), 2 μL of Primer F+R, 1 μL DNA template of putative fusions and parents and 9.5 μL ddH_2_O. The PCR procedure consisted of an initial denaturation at 94 °C for 5 min, followed by 38 cycles of 60 s at 94 °C, 90 s at 72 °C, and a final extension of 7 min at 72 °C. The amplified PCR products were analyzed via electrophoresis on 1% agarose gel and visualized. 

#### 2.4.3. Determination of Ergothioneine Content of Fusions and Parents

The fusions and their parents were cultured for 15 days f in PSB, and the mycelia were freeze-dried and weighed (marked as m_0_, g DW). The EGT content was analyzed by the method of Dubost et al. [29] with some modifications. Freeze-dried mycelia powder (0.1 g) of fusions and their parents was added with 8 mL of 70% ethanol and 2 mL of 1% SDS solution, after which it was kept at 4 °C for 20 h. After 10 ml of the solution described above was centrifuged at 25 °C and 8000 rpm for 10 min, the EGT content of the supernatant was determined by using the way of Xu et al. [30] with slight modification. The 10 mL supernatant was blown down to 3 mL using a nitrogen blower at 85 °C and the sample was fixed to 3 mL using tertiary water. In total, 1 ml of the above sample was filtered through a 0.22 μm aqueous microporous membrane and subjected to HPLC. The concentration of ergothioneine in the fusions and the parental mycelium was determined by utilizing a calibration curve generated from authentic standards and determining the values via HPLC analysis. The final result is presented as the ergothioneine content (EGT, mg/g DW) as calculated from the obtained data. The ergothioneine production in a 15-day liquid culture of fusions and parents was as follows:

$$P=EGT(mg/g)\times m_{0}\times 10$$

EGT: Content of ergothioneine in dry mycelium;

m_0_: Dry weight of mycelium obtained after 15 days of culture

### 2.5. Biological Characteristics of Fusions and Parents

#### 2.5.1. Determination of the Mycelium Growth Rate of Fusions and Parents

Mycelia blocks (5 mm diameter) derived from fusions and the original strains, in conjunction with the medium, were introduced to the middle of plates containing PDA and incubated at 28 °C. Three parallels were set for each temperature, and the colony diameter was measured every 1–2 days until the mycelium covered the Petri dish, and the number of days was recorded.

#### 2.5.2. Cultivation Characteristics of Fusions and Parents

The parental and all fusions strains were inoculated into 100 mL PSB medium at 5% inoculum and cultured at 28 °C and 150 rpm for 10 days as the seed liquid. The parental and fusions’ seed liquids were inoculated into 10 bags of cultivation medium with an inoculum of 3%, and the control quantity of dry substrate used was 230 g (dry weight). With the humidity controlled at 75–80%, the mycelium growth stage was cultivated in the dark at 24 °C and the fruiting stage was cultured at 24 °C in light. Mature fruiting bodies of fusions and their parents were collected, weighed and recorded. The biological efficiency (%) was determined using the following formula: BE (%) = (Fresh yield of fruiting body/Dry weight of substrate used) × 100% [31].

#### 2.5.3. Determination of Ergothioneine Content in Fusions and Parents

The fruiting bodies of *Ganoderma.* fusions and their parents were cut into pieces, frozen at −20 °C for 12 h, and then frozen in liquid nitrogen, ground and crushed, and passed through a 60-mesh sieve to collect the fruiting body powder and stored in a dry box.

The EGT content of the fruiting body was analyzed by using the method in Section 2.4.3 with sight modification. Fruiting body powder (0.5 g) of fusions and their parents was added with 16 mL of 70% ethanol and 4 mL of 1% SDS solution, after which it was kept at 4 °C for 20 h. After 20 mL of the solution described above was centrifuged at 25 °C and 8000 rpm for 10 min, 10 mL supernatant was used to determine the EGT content by using the way in Section 2.4.3. The EGT content of samples was determined by using HPLC as described above. The formula was as follows:

EGT(mg/g)=(C∗3∗2)/0.5

C: Content of EGT in HPLC sample

### 2.6. Re-Sequencing Analysis of Ganoderma Fusions 

#### 2.6.1. DNA Extraction and Sequencing

In order to investigate the differences in the synthesis of EGT in parental and fusions mycelium, resequencing of the total genomic DNA obtained in Section 2.4.2 was conducted on the parental strain FQ23 and the fifth-generation fusions RS7, RS8, and RS12 due to their varying levels of EGT content, respectively. The experimental procedure followed the protocol provided by Illumina. DNA libraries of 350 bp for Illumina/BGI sequencing were constructed for each accession. The DNA library was sequenced using an Illumina HiSeq XTen/NovaSeq/BGI platform by a commercial service provider, Biomarker Technologies located in Beijing, China with a read length of 150 bp. To guarantee the integrity of the sequencing data, a series of quality control measures were implemented. These included the elimination of pair-end reads containing more than 10% undefined bases, the removal of reads displaying a majority of bases with a quality score below 20, and the elimination of any remaining sequencing adapters. This resulted in obtaining high-quality sequences for further analysis.

#### 2.6.2. SNP and InDel Calling

The process of aligning the clean reads of each accession to the reference genome was carried out utilizing the Modified EM algorithm of the Burrows–Wheeler Aligner (BWA version 0.7.10-r789) [32]. Subsequent to sorting the mapping data, duplicate reads were identified and removed using the SAMTOOLS (v1.3.1) [33] and PICARD (v1.94) software programs. In order to enhance the precision of the mapping, a further step of local realignment was executed around regions of InDels using InDel-Realigner in GATK (v3.8) [34]. SNPs and InDels were identified among the four accessions using the HaplotypeCaller module in GATK. The identified variants were then filtered based on specific criteria, including: QD < 2.0 || MQ < 40.0 || FS > 60.0 || QUAL < 30.0 || MQrankSum < −12.5 || ReadPosRankSum < −8.0 -clusterSize 2 -clusterWindowSize 5. These filtered data were annotated using SnpEff software (v4.5) to determine the location of the variant loci and the predicted impact of the variations [35]. Further analysis was conducted by comparing the variant genes with functional databases, such as GO [36], COG [37], and KEGG [38] using BLAST [39]. The aim of this comparison was to identify genes with functional differences between the fusion strains RS7, RS8, and RS12 and the parental strain FQ23 based on the location of the variant loci and the gene location information on the reference genome.

## 3. Results

### 3.1. Number and Regeneration Rate of Protoplasts

**Figure 1:**
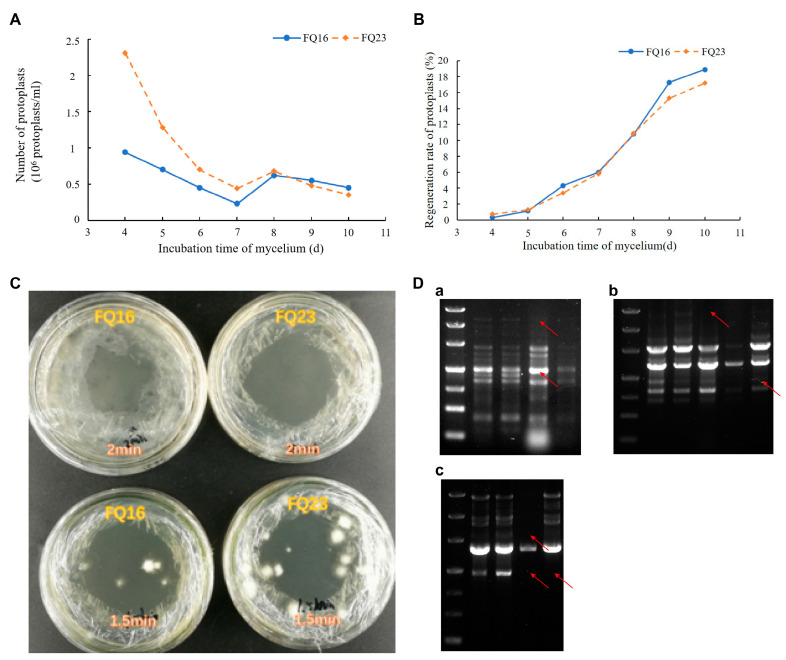
(**A**) The number of protoplasts. (**B**) Regeneration rate of protoplasts (**C**) The results of inactivation of protoplast exposed under UV for 2 min and 1.5 min. (**D**) ISSR profiles of fusions and parents. (**a**) Primer 864, (**b**) Primer 844, (**c**) Primer 825. Red arrows depict band deletion and blue arrows depict band increase.

The age of mycelium plays a significant role in determining the quantity and quality of prepared protoplasts and their subsequent regeneration rates [40]. Therefore, in order to obtain protoplast with high quantity and quality, mycelium cultured for 4, 5, 6, 7, 8, 9, and 10 days were utilized for protoplast preparation. As shown by Figure 1A, the number of protoplast preparations exhibited a declining trend with advancing mycelium age, whereas the rate of protoplast regeneration displayed an ascending pattern. In accordance with Figure 1B, mycelium aged 10 days was observed to yield the highest protoplast regeneration rates for both FQ23 and FQ16, with rates of 16% and 18%, respectively. Consequently, 10 days for mycelium culture was selected for protoplast protoplast regeneration.

### 3.2. Results of UV Inactivation and Labeling

As observed in Figure 1C, the protoplasts of FQ23 and FQ16 did not achieve successful regeneration after 2 min of inactivation with a 38 W UV lamp, and 1.5 min of inactivation could not achieve 100% inactivation. Consequently, the condition of UV exposure for 2 min was employed as the inactivation method of fusion protoplasts for FQ23 and FQ16.

### 3.3. Results of Ganoderma Fusion Identification

#### 3.3.1. The Antagonism Tests

The results of the antagonism test are shown in Figure S1. After the fifth generation, putative fusions (recorded as RS6, RS7, RS8, RS10, RS11, RS12 and RS14) exerted strong antagonistic interactions with their parental strains. This is evident from the distinct demarcation lines formed on the culture plates, indicating the stable genetic attributes and genetic divergence of the fusions from their parental strains [41]. The proposed fusion, RS14, exhibited conspicuous antithetic lines with the parents in the first three generations of subculture. However, the antithetic line between the fourth generation and FQ16 was gradually blurred and ultimately disappeared in the fifth generation. This observation suggests that the proposed fusion RS14 might not have achieved complete genetic fusion, with the possibility of some of its genetic traits being lost during subsequent subcultures.

#### 3.3.2. Results of ISSR Primer Screening

From a total of 82 primers, primers with good genomic polymorphism and high stability were scored as primers for ISSR-PCR, and the results of ISSR-PCR screening primers are shown in Table 1.

#### 3.3.3. Amplification of Genome of Fusions by ISSR PCR

The proposed fusions were identified by PCR employing the screened ISSR primers and the results are depicted in Figure 1D. Specifically, with primer 864, RS7 exhibited a significant band deletion in the 800 bp–1200 bp while RS11 displayed a band deletion spanning the 3000 bp–4500 bp. This differential banding pattern strongly indicated that the genotypes of the two proposed fusions significantly diverged from those of FQ23 and FQ16 (Figure 1(Da)). In the case of primer 844, RS6 exhibited a notable band deletion in the 3000 bp–4500 bp, RS8 showed multiple band deletion compared with the parents, and RS12 showed significant band deletion in the region of 500 bp–1200 bp. This observation suggested that the genomes of all three proposed fusions were significantly different from those of FQ23 and FQ16 (Figure 1(Db)). For primer 895 and 825, RS10 displayed a displayed deletion band at 2000 bp–3000 bp, and both RS10 and RS14 (Figure 1(Dc)) exhibited a significant deletion band at 1200 bp. To summarize, the proposed fusions above represented entirely novel genetic entities arising from the process of protoplast fusion.

#### 3.3.4. Ergothioneine Content in Fusions and Parents

Table 2 shows the EGT contents in fusions with FQ23 and FQ16 as references. The mycelium of fusion RS7 exhibited the highest total EGT content, measuring 2.273 ± 0.063 mg/g DW, surpassing both parental strains and demonstrating significant divergence from the other fusions. In contrast, the mycelium of RS14 contained the lowest EGT content at 0.932 ± 0.146 mg/g DW. FQ23, one of the parental strains, possessed the second-highest EGT content at 1.984 ± 0.015 mg/g, but exhibited the lowest dry weight of 0.460 ± 0.014 g after 15 days of liquid culture. On the other hand, the mycelium of fusion RS6 displayed a lower EGT content at 1.104 ± 0.443 mg/g but had the highest dry weight of 0.766 ± 0.093 g. This observation indicated that the selection of strains with high EGT synthesis capacity should take into account not only the EGT content of the mycelium but also the growth rate of the strain.

Upon evaluating the total EGT content following a 15-day liquid fermentation period (Table 2), it was observed that the most substantial EGT synthesis occurred in RS7, reaching 12.70 ± 1.85 mg/L. This represented a remarkable increase of 34.23% and 39.10% when compared to the EGT yields in FQ16 and FQ23, respectively. In contrast, RS10 exhibited the lowest EGT production at 5.34 ± 1.06 mg/L. The production of EGT in RS12 and RS14, while significantly distinct from that in FQ16 and FQ23, remained at lower levels. As analyzed above, RS7 not only boasted the highest EGT content in the mycelium among the fusions, but also demonstrated a high mycelial growth rate during liquid fermentation. Consequently, RS7 emerges as the preferred target strain.

### 3.4. Biological Characteristics of Fusions and Parents

#### 3.4.1. Results of Mycelial Growth Stage

The difference between the fusion and parent strains on the growth rate, the timing of reaching the full bag, and the degree of feeding were analyzed using Excel software (v2021) and SPSS 21.0. The results indicated that among all the strains, RS7 displayed the highest mycelial growth rate, measuring 0.83 ± 0.07 cm/day, followed by RS6 at 0.79 ± 0.12 cm/day. In contrast, RS14 exhibited the lowest growth rate at 0.63 ± 0.08 cm/day. No significant differences in full bag time or degree of feeding were observed among FQ16, FQ23, RS6, RS7, RS10, RS11, and RS12.

#### 3.4.2. Results of Fruiting Body Growth Stage 

The cultivation results (Table 3) indicated that there was no statistically significant difference in terms of fresh weight or biotransformation rate between the two parental strains. In contrast, among the fusions, RS14 displayed the lowest fresh weight of 36.68 ± 7.11 g, while RS8 demonstrated the highest fresh weight of 48.16 ± 7.18 g. Notably, RS6, RS7, RS8, RS10 and RS12 all demonstrated an average weight surpassing that of the two parent strains. These findings suggested an enhancement in their biotransformation capacities concerning cottonseed shells when compared to the parental strains.

Figure S2 illustrates the growth of fruiting bodies in the *Ganoderma* fusions and parent strains. As shown in the figure, there are significant differences in the morphology of the fruiting bodies in the fused strains when compared to the parents. Among the fusions, the cap of RS6 displayed limited expansion and was unable to reach full expansion. In contrast, RS7 exhibited the most extended cap with a distinct, dark, glossy coloration. RS8, RS10, RS11, RS12 and RS14 had lighter cap colors ranging from light brown to pale. Many of the fusions retained specific characteristics reminiscent of the parent strains, such as the fan-shaped fruiting bodies of RS6 and RS14, resembling those of FQ16, and the kidney-shaped morphology of RS7, RS8, RS10, RS11, and RS12, which bore a resemblance to the characteristics of FQ23.

#### 3.4.3. Determination of EGT Content in Fruiting Body of Fusions and Parental Strains

The EGT content in the fruiting body of successfully cultivated *Ganoderma* fusions was determined and analyzed by using SPSS 21.0, and the results are shown in Table 3. The results revealed that EGT was undetectable in the substrates of FQ23, RS7, and RS8. Among the remaining fusion strains, RS11 exhibited the highest EGT content of 12.24 ± 1.96 μg/g, followed by RS14 with 10.20 ± 1.83 μg/g. Notably, these EGT yields represented significant increases of 53.58% and 27.98%, respectively, when compared to FQ16. These strains displayed notably elevated EGT production in comparison to their parental strains, establishing them as promising new strains of *Ganoderma* spp. with high EGT production potential.

### 3.5. Results of Re-Sequencing of Ganoderma Fusions

#### 3.5.1. Annotation and Functional Analysis of SNPs and InDels

**Figure 2:**
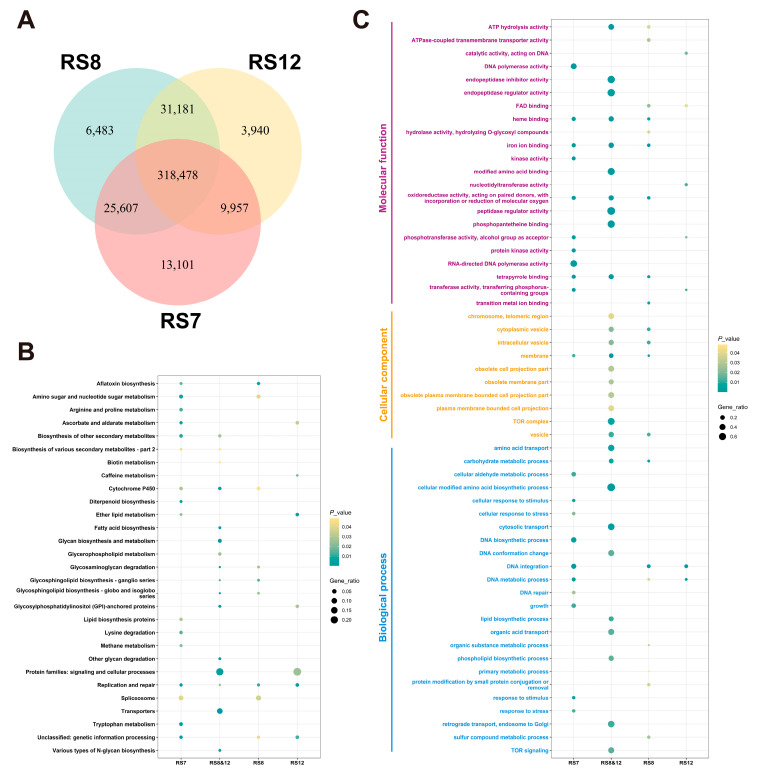
Annotation results of SNPs of fusions. (**A**) Comparison of the number of mutations in SNPs of fusions. (**B**) Summary of KEGG data annotation types for SNPs-associated genes of fusions. (**C**) GO classification of SNPs.

The mean genome resequencing coverage of the three fusions was 99.79%, 98.90%, and 95.28% at 1×, 5×, and 10× coverage depths, respectively. A total of 367,143, 381,776, and 363,585 SNPs and 51,465, 54,415, 50,083 InDels was identified by comparing the fusions RS7, RS8 and RS12 with the parental strain FQ23. GO classification and KEGG enrichment analysis of the variant loci in the CDs region with a corrected *p*-value < 0.05 was considered as an enrichment term. GO classification (Figure 2C) showed that the SNPs and InDels variant loci of the fusion strains were mainly enriched in molecular function, cellular component and biological process. Specifically, variant genes in the cellular component category were mainly classified in the membrane and ion binding categories, while those in the molecular function category were mainly classified in iron binding, reductionist activity, protein kinase activity, and transferred activity. In terms of physiological progress, many genes were found to be associated with amino acid anabolism and DNA metabolic processes and catalytic categories.

From Figure 2B, the darker blue color represents a larger proportion of SNPs within a particular class, and the larger bubbles represent a larger number of SNPs being involved. Compared to the other two fusions, RS7 exhibits a higher proportion of SNPs related to secondary metabolites. Among the SNPs unique to RS8, there are more variations in genes related to carbohydrate metabolism, potentially influencing overall nutrient uptake. In contrast, there are no major classes of SNPs were prominent in RS12-specific-SNPs, suggesting that the effect of SNPs on EGT synthesis can be excluded.

**Figure 3:**
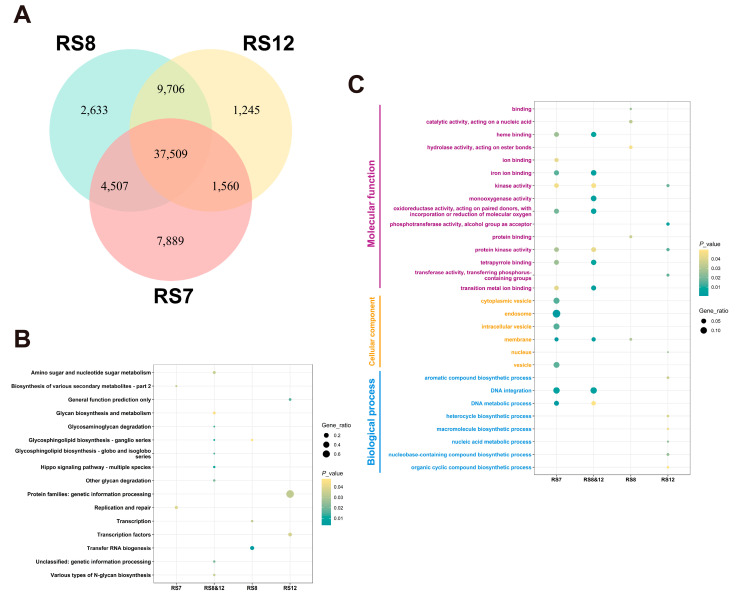
Annotation results of InDels of fusions. (**A**) Comparison of the number of mutations in InDels of fusions. (**B**) Summary of KEGG data annotation types for InDels-associated genes of fusions. (**C**) GO classification of InDels.

From Figure 3B, it is evident that RS8 and RS12 exhibit a higher proportion of InDels involved in carbohydrate anabolism compared to RS7-specific InDels, which may contribute to the decrease in EGT production. The distinction between RS8 and RS12 is that RS12 has a greater number of InDels affecting secondary metabolites and amino acid transport metabolism. The reduced EGT production in RS12 may be more strongly influenced by changes in small molecule metabolism, while the reduction in EGT in RS8 may be attributed to alterations related to the extensive class of nucleotides involved in replication, transcription and nucleotide modifications.

#### 3.5.2. Annotation and Functional Analysis of Genes with Copy Number Changes

**Figure 4:**
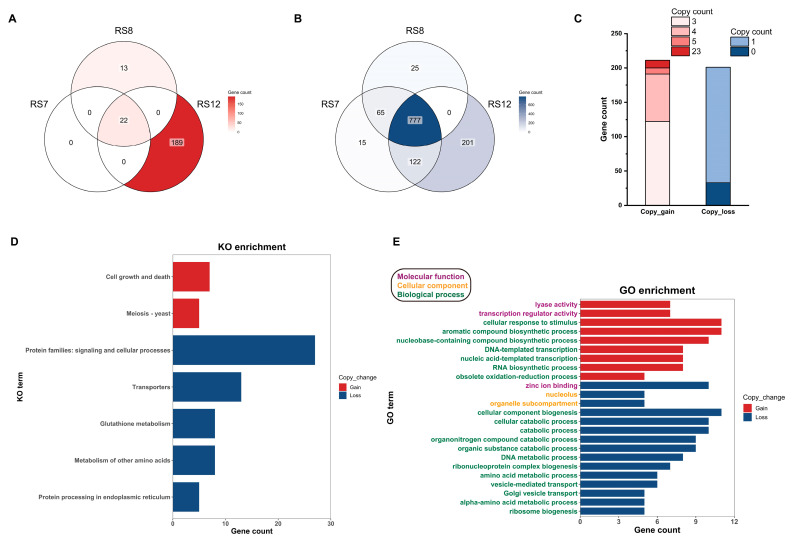
Comparative analysis of fusions’ copy number. (**A**) Comparison of copy number reduction. (**B**) Comparison of copy number increase. (**C**) Gene copy number changes in RS12. (**D**) KO enrichment classification of gene with copy changes in RS12. (**E**) GO enrichment classification of gene with copy changes in RS12.

The differences in gene copy numbers between fusions are illustrated in Figure 4A,B. Notably, the increased copy number in RS7 was found in only 22 genes, and these genes were also present in RS8 and RS12, although they were not included in the analysis. The copy number changes in RS8 were limited, with only 13 increased sites and 25 decreased sites, suggesting a minimal impact on RS8. In contrast, RS12 displayed significant variations in gene copy numbers, with 189 genomic fragments showing an increase in copy number and 201 experiencing a decrease. Given the relatively low number of SNPs and InDels in RS12, it can be inferred that changes in gene copy numbers were the primary contributing factor to the decrease in EGT synthesis in RS12.

As shown in Figure 4, enrichment analysis of the KEGG and GO databases for genes involved in copy number changes in RS12 fusions showed that genes with increased copy number had a significant impact on processes related to growth and proliferation (Figure 4D), as well as regulation of genetic material (Figure 4E). In contrast, the genes with reduced copy number were associated with signaling and material transport, as well as in the anabolism of various small molecule compounds. Consequently, it is speculated that the copy number changes in the RS12 fusion affect overall biosynthesis, as more nutrients are directed toward division and proliferation and less toward substance synthesis. This shift could lead to higher biomass and a decrease in the production of EGT, a secondary metabolite, due to the increased use of precursor amino acids for reproduction.

Among the RS12-specific genes, there are gene fragments with copy numbers as high as 23 (Figure 4C), which involve two key genes, methionyl aminopeptidase (EVM0002287) and cellulose growth-specific protein (EVM0008869). The elevated copy number of these genes may contribute to the enhanced mycelium growth and division observed in RS12. Additionally, RS12 exhibited a decrease in copy numbers of GST genes related to glutathione metabolism. This reduction may lead to a decreased efficiency in glutathione metabolism, resulting in the accumulation of precursor compounds such as γ-glutamylcysteine (γ-Gln-Cys). The elevated levels of γ-Gln-Cys levels could facilitate the biosynthesis of Her-Cys-Gln, which cannot convert to ergothioneine in edible fungus, leading to a decrease in ergothioneine synthesis [42].

#### 3.5.3. Effect of Gene Mutation on the EGT Synthesis Pathway

**Figure 5:**
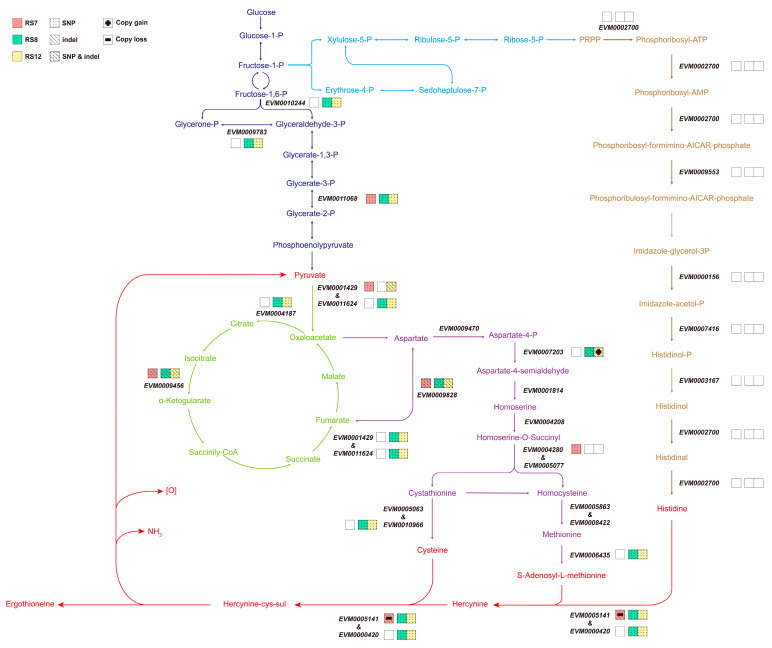
Map of metabolic pathways involved in EGT synthesis by the mutated genes occurring in each fusion.

Considering the distinct biological characteristics of the fusions RS7, RS8, and RS12, with RS7 exhibiting elevated EGT production, it was selected as a reference for comparison with RS8 and RS12, both of which displayed reduced EGT production. In this comparative analysis, the genomic mutations present specifically in RS7, as well as those present in either RS8 or RS12, were analyzed with the aim of identifying the key mutations potentially responsible for the observed differences in EGT production. As shown in Figure 5, the biosynthesis of EGT involves the synthesis of histidine, methionine, and cysteine, with histidine produced via the pentose phosphate pathway and methionine and cysteine produced by the TCA cycle. The resequencing results indicated that the three fusions did not produce separate mutations in the genes responsible for the histidine synthesis pathway (PRPP as the source), effectively excluding the possibility of EGT production alteration stemming from mutations in the histidine synthesis pathway. The biosynthesis of methionine and cysteine originates from the same amino acid, namely aspartic acid. On the allosteric pathway of aspartate, RS8 and RS12 exhibited distinct SNPs and InDels mutations in the EMV0007203 gene, while RS12 additionally showed an elevated copy number. Mutations in this gene are likely to exert a significant effect on methionine and cysteine, contributing to the reduction in EGT production. In addition, RS8 and RS12 displayed SNP mutations in EMV0010966 and EMV0006435, which could affect the synthesis of cysteine and S-Adenosy-L-methionine (SAM). Within the tricarboxylic acid cycle, SNPs and InDels were observed in the synthesis of oxaloacetate from pyruvate, which could affect the production of oxaloacetate (an aspartate precursor).

As depicted in Figure S3, the gene EVM0005141 was annotated to possess a specific deletion mutation in RS7. This deletion resulted in a shift mutation in the 1009-base reading frame, leading to a modification and premature termination of subsequent transcription and translation processes. An analysis of the amino acid sequences before and after the mutation, based on the NCBI database, revealed that the EVM0005141 gene of FQ23 encodes two structural domains of the protein: a DinB-2 domain near the N-terminus that helps to capture histidine and react with metal ions and an egtB-related domain that promotes the formation of C-S bonds. The mutation of the EVM0005141 gene in RS7 caused a forward shift of the stop codon, resulting in the shortening of the whole protein sequence. This led to the presence of only FGE-sulfatase in the C-terminal structural domain. However, this altered protein structure could not execute its intended function due to the incomplete sequence, ultimately culminating in the absence of egtB synthesis function in RS7.

**Figure 6:**
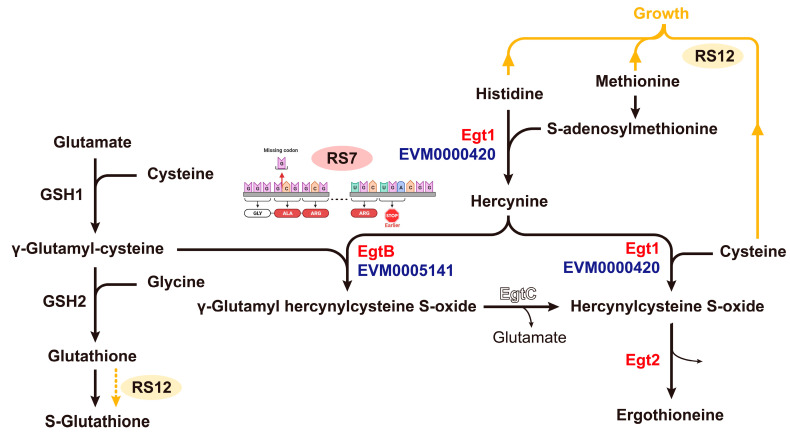
Schematic diagram of the EGT synthesis pathway before and after gene mutation in fusions.

*Ganoderma* spp. is a filamentous fungus that shares common EGT synthesis genes with other fungi such as *N. crassa*, *G. frondosa*, and *Pleurotus ostreatus* [43]. Specifically, the gene EVM0005141 is annotated with a structural domain related to histidine methylation and C-S binding (egtB). In contrast, the gene EVM0000420 possesses two complete structural domains (Figure 6), similar in structure to other reported EGT1 genes [44]. The EVM0005141 gene, according to the comparison results, exhibits a distinctive consecutive amino acid sequence YY. However, the amino acid sequence of this gene lacks this characteristic of Type II EgtB, thus indicating it to be Type I EgtB described by Kamide et al. [45]. This suggests that it is a type I C-S binding gene, which requires histidine betaine and γ-glutamylcysteine to form the C-S bond. On the other hand, the gene EVM0000420 is a type II C-S binding gene, requiring only amino acid betaine and cysteine to complete the C-S bond.

Given that glutathione synthesis is present in filamentous fungi, the intermediate product γ-Glutamylcysteine is naturally produced. If egtB (EVM0005141) performs the corresponding enzymatic function, it will facilitate the formation of C-S bonds between Hercynine and γ-Glutamylcysteine, ultimately yielding γ-Glutamyl hercynylcysteine S-oxide [46]. The subsequent conversion of this compound to Hercynylcysteine S-oxide requires the involvement of egtC, which is responsible for cleaving the glutamyl group. However, there are no genes with egtC structural domains reported in the research among fungi, and no known enzymes capable of directly cleaving the C-S bond between histidine betaine cysteine and γ-glutamyl cysteine. Therefore, mutations in the RS7 EVM0005141 gene and copy number deletions result in a reduction in the nonsense shunt involving histidine betaine (Figure 6). This reduction, in turn, enhances the synthesis of EGT.

## 4. Discussion

The antagonistic test serves as a rapid and efficient method to identify fusion strains; nevertheless, it does have inherent limitations. In this study, the confrontation line between RS14 and FQ16 began to blur from the fourth generation, indicating that RS14 was not a genetically stable fusion. Nonetheless, the ISSR-PCR results revealed distinct genomic disparities between RS14 and both parental strains FQ16 and FQ23. These discrepancies were not evident in the antagonism test. Therefore, the antagonism test may not accurately distinguish strains that are too closely related. Consequently, it is advisable to complement the antagonism test with molecular biological methods for precise strain identification in such cases.

The phenomenon of nuclear transfer after cell fusion has been reported. Park et al. [47] investigated the phenomenon of nuclear transfer after the fusion of protoplasts of *Ganoderma lucidum* and *Coriolus versicolor*. Their investigation revealed that the fusions exhibited isozyme bands from both parent strains in different amounts, along with some novel isozyme bands identified by isozyme profiles. Moreover, DNA analysis confirmed that these strains were diploid, inheriting genetic material from both parental strains. Kim et al. transferred nuclei from *Lentinula edodes* into protoplasts of *Coriolus versicolor* and studied the number of nuclei per protoplast and the amount of DNA per nucleus. This investigation revealed varying DNA contents among the hybrids, while the number of nuclei per cell remained consistent. Notably, certain hybrids exhibited over three times the DNA content of the parent strains, substantiating the existence of nuclear hybridization [48]. Dudits et al. [49] investigated the phenomenon of gene transfer after the fusion of parsley and carrot. Their cytological studies demonstrated that the number of chromosomes in fusions was different from that of its parents, and both parental genotypes were partially retained in the fusion. It has been also reported that in stable mammalian fusion cells, the genotypes of the two parents are integrated, while in unstable fusion cells, they remain separate. Furthermore, instability and deletions in the genotypes have been observed following gene transfer [50,51]. This process eventually gives rise to fusions that inherit a combination of genotypes from both parent strains in addition to generating new genotypes resulting from DNA recombination. It is important to note that this process does not typically lead to the formation of triploid or polyploid cells.

In summary, the stable genetic growth of fusions following the fusion and recombination of parental genomes signifies a successful recombination process. In contrast, fusions that primarily retain parental genomes tend to exhibit instability and are difficult to regenerate. The chromosome number of the fused progeny may differ from that of the parents, retain part of the parental traits, or even produce new genotypes or gene loss. It is hypothesized that the blurring of the confrontation line between RS14 and FQ23 during transmission may be attributed to the loss of specific genes from FQ23 during protoplast fusion, leading to the loss of certain traits of FQ23 in RS14 and an increased affinity of RS14 for parental FQ16, resulting in a blurred confrontation line. Further experimentation is needed to confirm this hypothesis.

Lai et al. [52] obtained new strains with high production of cordycepin and cordycepic acid by fusing protoplasts of *Antrodia cinnamomea* and *Cordyceps militaris*; Peng et al. [53] conducted protoplast fusion involving two *Ganoderma lucidum* protoplasts and obtained a new strain of *Ganoderma* with high production of polysaccharides and triterpenes. From the above research, it is clear that protoplast fusion is a common breeding tool within the field of *Ganoderma* breeding. It offers a practical means to obtain new strains characterized by elevated yields of specific bioactive compounds or strains that amalgamate favorable attributes from two parent strains. We utilized protoplast fusion to obtain new strains characterized by increased EGT production compared to their parent strains. Additionally, we acquired fusions that displayed diverse characteristics. The results indicate that fusions obtained by protoplast fusion are stochastic, and the new genetic traits resulting from gene recombination can produce a variety of results. For instance, the targeted fusion RS7, distinguished by its high EGT production, may be attributed to mutations within EVM0005141, while fusion RS8 exhibited higher substrate production and fusion RS11 displayed an enhanced EGT production in the fruiting body.

Lee et al. [54] conducted a comparative analysis of EGT content within the mycelia and fruiting body of eight macrofungi, including *G. neo-japonicum* and *G. applanatum*. The study revealed that *Ganoderma neo-japonicum* had the highest EGT content in mycelia, approximately tenfold greater than that observed in its fruiting bodies. Cohen et al. [55] examined the EGT content in *Ganoderma lucidum* mycelia and fruiting bodies and found that EGT was not detected in substrates but was successfully detected in mycelia. Lo et al. [56] examined the EGT content of *G. lucidum* mycelia and fruiting bodies, revealing that the EGT content of fruiting bodies was approximately three times lower than that of mycelia. The collective evidence from these studies consistently suggests that EGT content tends to be higher in *Ganoderma* mycelia compared to their fruiting bodies. Therefore, in this study, *G. resinaceum* strain FQ23 and *G. sessile* strain FQ16 with high EGT production in mycelia were selected as parents and used for protoplast fusion to obtain new strains with high EGT production. Following the selection and identification of the fusions, the EGT content of RS7 mycelium surpassed that of the two parent strains. Furthermore, RS7 displayed mycelial growth capabilities intermediate to those of the parent strains when cultivated in a liquid medium. In summary, the total amount of EGT obtained from RS7 for 15 days of liquid culture surpassed that of the two parents. Consequently, RS7 was identified as the target strain with high EGT production obtained in this study. Moreover, RS11 displayed significantly elevated EGT content in the fruiting body and maintained comparable mycelial growth rates to their parental strains. These characteristics designate RS11 as a promising candidate for elevated EGT production in fruiting bodies of *Ganoderma* spp.

In conclusion, seven new *Ganoderma* fusions with good genetic stability were successfully generated in this study. Among these, RS7 emerged as a newly identified strain distinguished by its remarkable capacity for high EGT production in the mycelium. Concurrently, RS11 introduced a novel *Ganoderma* spp. strain recognized for its heightened EGT production within the fruiting bodies. Additionally, the resequencing of RS7, RS8, and RS12 shed light on the intricate interplay between ergothioneine synthesis and various metabolic pathways in *Ganoderma* strains. These findings not only provide valuable insights for breeding strategies but also furnish essential theoretical guidance for the future development of superior *Ganoderma* strains.

## Figures and Tables

**Table 1:** Details of ISSR primers used in this study.

Primer Name	Sequence (5′-3′)	Tm (°C)
810	GAGAGAGAGAGAGAGAT	49
827	ACACACACACACACACG	51
844	CTCTCTCTCTCTCTCTRC	52
846	CACACACACACACACART	50
850	GTGTGTGTGTGTGTGTYC	52
855	ACACACACACACACACYT	50
856	ACACACACACACACACYA	50
858	TGTGTGTGTGTGTGTGRT	50
860	TGTGTGTGTGTGTGTGRA	50
864	ATGATGATGATGATGATG	45
865	CCGCCGCCGCCGCCGCCG	72
867	GGCGGCGGCGGCGGCGGC	72
868	GAAGAAGAAGAAGAAGAA	45
887	DVDTCTCTCTCTCTCTC	47

**Table 2:** Dry weight of mycelium and its total ergothioneine synthesis in 15-day liquid cultures of fusions and parents.

Trial Code	Dry Weight (g)	EGT in Mycelium (mg/g DW)	Total EGT Synthesis (mg/L)
FQ16	0.637 ± 0.023 ^abc^	1.467 ± 0.015 ^abcd^	9.51 ± 0.38 ^c^
FQ23	0.460 ± 0.014 ^a^	1.985 ± 0.020 ^cd^	9.13 ± 0.21 ^c^
RS6	0.766 ± 0.093 ^c^	1.105 ± 0.443 ^ab^	8.25 ± 2.37 ^bc^
RS7	0.561 ± 0.096 ^ab^	2.273 ± 0.063 ^c^	12.70 ± 1.85 ^d^
RS8	0.509 ± 0.060 ^ab^	1.499 ± 0.299 ^bcd^	7.41 ± 0.26 ^bc^
RS10	0.507 ± 0.061 ^ab^	1.046 ± 0.081 ^ab^	5.34 ± 1.06 ^ab^
RS11	0.514 ± 0.021 ^ab^	1.367 ± 0.046 ^abc^	7.02 ± 0.18 ^bc^
RS12	0.605 ± 0.022 ^abc^	1.081 ± 0.281 ^ab^	6.39 ± 1.56 ^abc^
RS14	0.708 ± 0.067 ^bc^	0.932 ± 0.146 ^ab^	6.28 ± 1.28 ^abc^

Significant differences (*p* < 0.05) in mycelium EGT content between strains are indicated using (a, b, c and d).

**Table 3:** Comparison of agronomic traits of fusions and parents.

Strain Number	Mycelium Growth Rate (cm/d)	Full Bag Days (d)	Fresh Weight of Fruiting Body/g	Biotransformation Rate (%)	EGT in Fruiting Body (μg/g)
FQ16	0.71 ± 0.06 ^abcd^	15.5 ± 0.5 ^a^	38.56 ± 5.04 ^bcde^	16.76 ± 2.19	7.97 ± 0.81 ^ab^
FQ23	0.75 ± 0.80 ^bcd^	14.6 ± 0.7 ^a^	37.53 ± 2.56 ^bcde^	16.32 ± 1.11	/
RS6	0.79 ± 0.12 ^cd^	14.3 ± 0.9 ^a^	42.12 ± 1.67 ^cde^	18.31 ± 0.73	9.21 ± 0.76 ^abc^
RS7	0.83 ± 0.07 ^d^	14.2 ± 0.6 ^a^	42.70 ± 6.60 ^bc^	18.57 ± 2.87	/
RS8	0.69 ± 0.10 ^abc^	16.2 ± 2.4 ^ab^	48.16 ± 7.18 ^b^	20.94 ± 3.12	/
RS10	0.74 ± 0.05 ^bcd^	14.7 ± 0.7 ^a^	39.82 ± 6.24 ^de^	17.31 ± 2.71	10.06 ± 1.03 ^abc^
RS11	0.69 ± 0.09 ^abc^	15.6 ± 1.6 ^a^	37.27 ± 4.46 ^e^	16.21 ± 1.94	12.24 ± 1.96 ^cd^
RS12	0.72 ± 0.09 ^abcd^	14.8 ± 2.4 ^a^	42.41 ± 3.39 ^f^	18.44 ± 1.47	7.79 ± 0.59 ^ab^
RS14	0.63 ± 0.08 ^ab^	16.7 ± 1.9 ^ab^	36.68 ± 7.11 ^bcd^	15.95 ± 3.09	10.20 ± 1.83 ^bc^

Note: a, b, c, d, e and f indicate the significant difference in fresh weight of different strains of fruiting body (*p* < 0.05).

## Data Availability

The raw data that support the Illumina-sequencing analysis of this study are not openly available due to reasons of sensitivity and are available from the corresponding author upon reasonable request. Informed consent was obtained from all subjects involved in the study.

## Supplementary Materials

The following supporting information can be downloaded at: https://www.mdpi.com/article/10.3390/jof9111072/s1, Figure S1: Fusions from one to five generations of antagonism test (Ⅰ–Ⅴ represent the number of generations). Figure S2: Growth of fruiting body of fusions and their parental strains. Figure S3: Point mutation site information of gene EVM0005141 in RS7 compared to FQ23.
