# Supplementary material for: Ganoderma Fusions with High Yield of Ergothioneine and Comparative Analysis of Its Genomics
Source: J Fungi (Basel). 2023 Nov 2;9(11):1072. doi: 10.3390/jof9111072 (PMC10672712; doi:10.3390/jof9111072)
Supplement: Supplementary file 1 [file jof-09-01072-s001.zip › jof-2534068-supplementary.pdf]

Supplementary materials

Journal name: Journal of Fungi

Ganoderma Fusions with High Yield of Ergothioneine and Comparative Analysis of Its Genomics

Jiaqi Xie [1,2,†], Yinghao Yu [1,2,†], Junjiang You [1,2], Zhiwei Ye [1,2,*], Fenglong Zhou [1,2], Na Wang [3], Jingru Zhong [3], Liqiong Guo [1,2] and Junfang Lin [1,2,*]

1. College of Food Science, South China Agricultural University, Guangzhou 510640, China
2. Research Center for Micro-Ecological Agent Engineering and Technology of Guangdong Province, Guangzhou 510640, China
3. Guangzhou Alchemy Biotechnology Co. Ltd., 139 Hongming Road Guangzhou Economic Technology Zone, Guangzhou 510760, China

* Correspondence: zhwye@scau.edu.cn (Z.Y.); linjf@scau.edu.cn (J.L.); Tel.: +86-20-87570302 (Z.Y. & J.L.); Fax: +86-20-85280270 (Z.Y. & J.L.)

† These authors contributed equally to this work.

Supplementary data for Manuscript

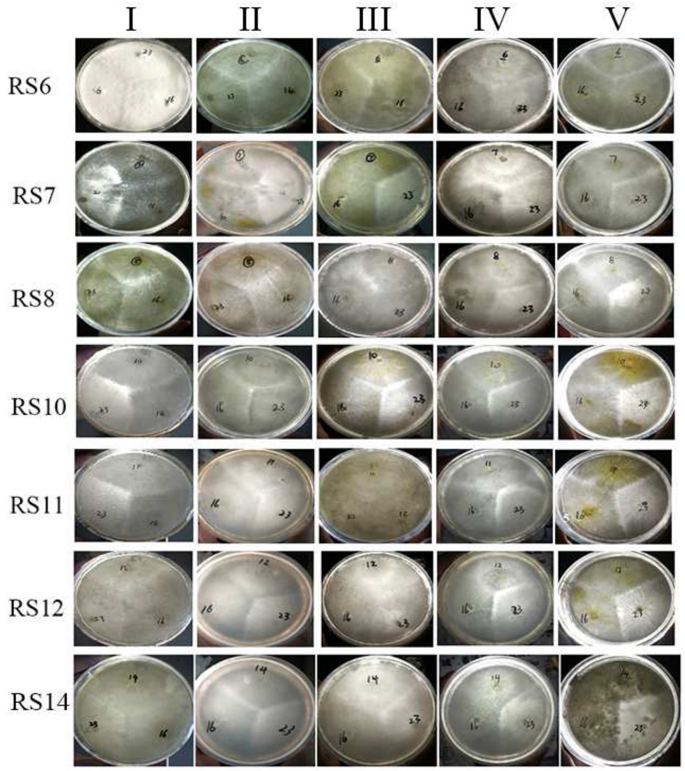

Figure S1 Fusions from one to five generations of antagonism test (I-V represent the number of generations)

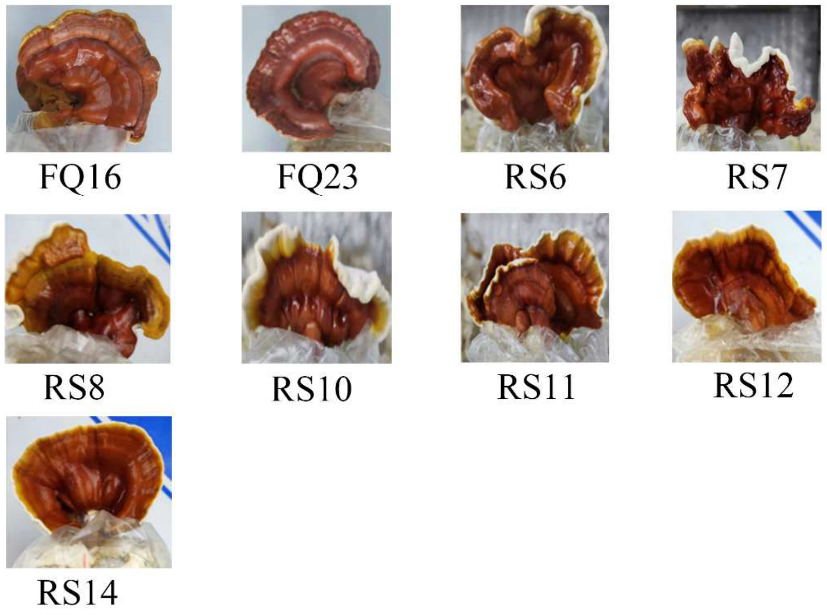

Figure S2 Growth of fruiting body of fusions and their parental strains

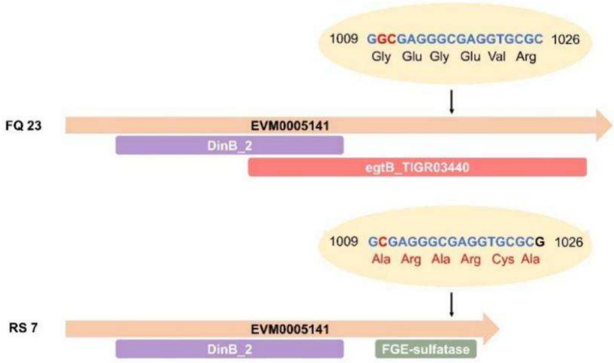

Figure S3 Point mutation site information of gene EVM0005141 in RS7 compared to FQ23
